# Statistical Classification Strategy for Proton Magnetic Resonance Spectra of Soft Tissue Sarcoma: An Exploratory Study with Potential Clinical Utility

**DOI:** 10.1080/1357714021000065396

**Published:** 2002-09

**Authors:** Tedros Bezabeh, Samy El-Sayed, Rakesh Patel, Ray L. Somorjai, Vivien Bramwell, Rita Kandel, Ian C. P. Smith

**Affiliations:** 1 Institute for Biodiagnostics National Research Council of Canada 435 Ellice Ave. Manitoba Winnipeg R3B 1Y6 Canada; 2 CancerCare Manitoba Winnipeg Canada; 3 London Regional Cancer Centre Ontario London Canada; 4 Department of Pathology and Laboratory Medicine Mount Sinai Hospital Ontario Toronto Canada

## Abstract

*Purpose:* Histological grading is currently one of the best predictors of tumor behavior and outcome in soft tissue sarcoma. However, occasionally there is significant disagreement even among expert pathologists. An alternative method that gives more reliable and non-subjective diagnostic information is needed. The potential use of proton magnetic resonance spectroscopy in combination with an appropriate statistical classification strategy was tested here in differentiating normal mesenchymal tissue from soft tissue sarcoma.

*Methods:* Fifty-four normal and soft tissue sarcoma specimens of various histological types were obtained from 15 patients. One-dimensional proton magnetic resonance spectra were acquired at 360 MHz. Spectral data were analyzed by using both the conventional peak area ratios and a specific statistical classification strategy.

*Results:* The statistical classification strategy gave much better results than the conventional analysis. The overall classification accuracy (based on the histopathology of the MRS specimens) in differentiating normal mesenchymal from soft tissue sarcoma was 93%, with a sensitivity of 100% and specificity of 88%.The results in the test set were 83, 92 and 76%, respectively. Our optimal region selection algorithm identified six spectral regions with discriminating potential, including those assigned to choline, creatine, glutamine, glutamic acid and lipid.

*Conclusion:* Proton magnetic resonance spectroscopy combined with a statistical classification strategy gave good results in differentiating normal mesenchymal tissue from soft tissue sarcoma specimens *ex vivo*. Such an approach may also differentiate benign tumors from malignant ones and this will be explored in future studies.


*Sarcoma* (2002) **6**, 97–103

Taylor & Francis healthsciences

**ORIGINAL ARTICLE**

### Statistical classification strategy for proton magnetic resonance spectra of soft tissue sarcoma: an exploratory study with potential clinical utility

TEDROS BEZABEH[1], SAMY EL-SAYED[2], RAKESH PATEL[1], RAY L. SOMORJAI[1], VIVIEN BRAMWELL[3], RITA KANDEL[4] & IAN C.P. SMITH[1]

[1]*Institute for Biodiagnostics, National Research Council of Canada, Winnipeg, Canada,* [2]*CancerCare Manitoba, Winnipeg, Canada,* [3]*London Regional Cancer Centre, London, Ontario, Canada,* [4]*Department of Pathology and Laboratory Medicine, Mount Sinai Hospital, Toronto, Ontario, Canada*

**Abstract**

*Purpose:* Histological grading is currently one of the best predictors of tumor behavior and outcome in soft tissue sarcoma. However, occasionally there is significant disagreement even among expert pathologists. An alternative method that gives more reliable and non-subjective diagnostic information is needed. The potential use of proton magnetic resonance spectroscopy in combination with an appropriate statistical classification strategy was tested here in differentiating normal mesenchymal tissue from soft tissue sarcoma.

*Methods:* Fifty-four normal and soft tissue sarcoma specimens of various histological types were obtained from 15 patients. One-dimensional proton magnetic resonance spectra were acquired at 360 MHz. Spectral data were analyzed by using both the conventional peak area ratios and a specific statistical classification strategy.

*Results:* The statistical classification strategy gave much better results than the conventional analysis. The overall classification accuracy (based on the histopathology of the MRS specimens) in differentiating normal mesenchymal from soft tissue sarcoma was 93%, with a sensitivity of 100% and specificity of 88%. The results in the test set were 83, 92 and 76%, respectively. Our optimal region selection algorithm identified six spectral regions with discriminating potential, including those assigned to choline, creatine, glutamine, glutamic acid and lipid.

*Conclusion:* Proton magnetic resonance spectroscopy combined with a statistical classification strategy gave good results in differentiating normal mesenchymal tissue from soft tissue sarcoma specimens *ex vivo*. Such an approach may also differentiate benign tumors from malignant ones and this will be explored in future studies.

### Introduction

In current clinical practice, the biological behaviour of soft tissue sarcoma tumors is best predicted on the basis of size and histological grade as determined by mitotic index, cellularity, necrosis, and the degree of nuclear anaplasia. Such parameters have been found to be useful prognostic indicators for survival in soft tissue sarcoma of the extremities.[1] However, in some cases there is significant disagreement among expert pathologists in the typing and grading of these tumors, and as a result, the accurate diagnosis of soft tissue sarcomas remains a clinical challenge.[2–4] Many of these tumors are quite large and only small fractions are sampled for histopathology. These limitations of histopathology, as well as the need to pursue new prognostic and treatment selection factors, provide the rationale for a search for more accurate and less subjective approaches in the diagnosis of soft tissue sarcoma.

New and advanced methods are becoming increasingly useful in identifying new prognostic markers that could guide the management of patients with soft tissue sarcoma. These include cytogenetic and molecular detection of chromosome translocation and gene fusions.[2,5,6] These techniques are still experimental and only available in a small number of centers. Another modality with enormous diagnostic potential is magnetic resonance spectroscopy (MRS).

To date, most of the magnetic resonance reports on soft tissue sarcoma have dealt with imaging, with little emphasis on spectroscopy. Magnetic resonance imaging (MRI) has proven to be more useful than CT because of its superior soft tissue contrast and multiplanar imaging capability, and it is now considered to be the imaging technique of choice for soft tissue masses.[7–9] It has been successfully used in radiotherapy treatment planning and tumor volume

Correspondence to: Dr. Tedros Bezabeh, Institute for Biodiagnostics, National Research Council, 435 Ellice Ave., Winnipeg, Manitoba, Canada R3B 1Y6; Tel.: +1-204-983-0994; Fax: +1-204-984-7036; E-mail: tedros.bezabeh@nrc.ca

ISSN 1357–714X print/ISSN 1369–1643 online/02/0200097–07 © Taylor & Francis Ltd
DOI: 10.1080/1357714021000065396

estimation in soft tissue sarcomas.[10] However, its diagnostic specificity remains inadequate. For example, MRI cannot reliably distinguish between benign and malignant soft tissue sarcomas — well differentiated liposarcomas have a high fat content and may resemble benign lipomas on MRI images. Moreover, inflammatory changes and hematoma could mimic a tumor in an MRI image. This, however, is a lesser problem with MRS.

Magnetic resonance spectroscopy has the ability to go beyond anatomical/morphological information and probe the biochemistry of tissues at the cellular level. Clinical issues regarding tumor aggressiveness, metastasis and recurrence may be addressed better by spectroscopic investigation, since the technique yields information on the biochemical and metabolic changes occurring in the tumor during its development and growth. Moreover, the problem of undersampling in MRS is not as much of a concern as it is in routine histopathological procedures. Early MRS work on soft tissue sarcoma focussed on $^{31}P$ MRS.[11–15] Such studies have generated useful information by revealing that spectra of tumors show high levels of phospholipids and inorganic phosphate, and low levels of phosphocreatine. In addition, proton-decoupled $^{13}C$ MRS study by Singer *et al.* has shown a correlation between the fatty acyl chain content and the histological type and grade of liposarcomas.[16]

The higher detection sensitivity of $^{1}H$ relative to $^{31}P$ and $^{13}C$ offers a significant advantage. However, the $^{1}H$ MRS studies performed on soft tissue sarcoma have so far focussed primarily on liposarcomas and lipomas. Recently, Singer *et al.* have shown that a significant correlation exists between the degree of unsaturation of fatty acyl chains, obtained from two-dimensional MRS, and the mitotic activity, indicator of grade or degree of differentiation, in soft tissue sarcomas.[17] However, such two-dimensional experiments require long acquisition times and are complicated to perform. Millis *et al.* in their recent work used both one- and two-dimensional approaches, but they limited their study to liposarcomas.[18,19] They found that the NMR-visible level of triglyceride correlated with liposarcoma differentiation, with the well-differentiated tumors having the highest level and the dedifferentiated and/or pleomorphic subtypes (most aggressive and metastatic subtype) having the lowest. It is worthwhile to point out here that their 1D data, performed with the use of magic angle spinning, were not analyzed by methods such as the one used herein.

Although liposarcomas are amongst the common types of soft tissue sarcoma, they represent only about 20% of all soft tissue sarcoma. Malignant fibrous histiocytoma (MFH), leiomyosarcoma, and fibrosarcoma together, account for about 50% of soft tissue sarcomas. Thus, some effort should also be directed towards the MRS study of such types of soft tissue malignancy to determine whether they share similar spectral characteristics.

One-dimensional spectra and a sophisticated statistical classification strategy (SCS) should provide a simple and more reliable approach to diagnosis. One-dimensional MR spectra contain a set of resonances whose chemical shifts, relative to a standard, are indicative of the nature of the biochemical and metabolic species responsible for them. The intensities of such resonances correspond to the relative amounts of the species generating the signals. Although the MR spectral data are rich in information of potential diagnostic and prognostic value, conventional methods of analysis fail to make complete use of this valuable information. Our statistical classification strategy, by virtue of its ability to utilize all the information in the spectra, provides a means of analyzing MRS data in a robust, non-subjective and reliable manner. This method has been successfully used in the classification of various other normal/benign and malignant tissue specimens, e.g., breast,[20] prostate,[21,22] colon,[23] brain,[24] ovary[25] and thyroid.[26]

The objective of this *ex vivo* study was to characterize the spectral features indicative of malignancy that are common to the various histological types of soft tissue sarcoma. Using the statistical classification strategy, we sought to develop a robust MRS-based classifier that can be reliably used in a clinical setting to differentiate between normal mesenchymal tissue and soft tissue sarcoma specimens.

### Materials and methods

Soft tissue sarcoma and adjacent normal tissue specimens from a tissue bank at Mount Sinai Hospital (Toronto), administered by the Canadian National Sarcoma Group, were employed in the study. The specimens were obtained in accordance with the Canadian Tri-Council Policy on the use of human tissue for medical research. Consent for the use of the excised tissue specimen (anonymized) was obtained from each patient. Table 1 shows the breakdown for the various cases. As can be seen, the major types of soft tissue sarcoma are well represented. Specimens from both the tumor and the surrounding normal tissue were provided for each case. The normal specimens were primarily composed of muscle (see Table 1).

Whenever possible, the specimens were divided into two or three pieces and spectra obtained from each piece. Thus, a total of 62 spectra (31 normal and 31 malignant) were acquired. However, the quality of eight spectra was considered non-optimal, and these spectra were excluded from the set before the data were subjected to the SCS. Hence, only 54 spectra (27 normal, 27 malignant) were included in the analysis (see Table 1).

The frozen specimens were thawed, mounted in small capillary tubes and subjected to MRS at 25°C as in Kuesel *et al.*,[27] using a high-resolution 360-MHz NMR spectrometer (Bruker Instruments, Billerica, MA). Acquisition parameters included:

**Table 1.** *Histological data of the soft tissue specimens included in the study*

| Specimen | Normal | Malignant |
|---|---|---|
| 419.001 | Muscle | — |
| 419.002 | Fat, 20% muscle | — |
| 420.001 | — | MFH |
| 420.002 | — | MFH |
| 433.001 | Muscle | Liposarcoma |
| 433.002 | Muscle, 20% fibrous tissue/fat | Liposarcoma |
| 438.001 | Muscle | Leiomyosarcoma |
| 438.002 | 90% muscle, 10% fat | Leiomyosarcoma |
| 438.003 | Muscle | — |
| 445.002 | — | * |
| 450.001 | 75% muscle, 25% fat and fibrous tissue | MFH |
| 450.002 | Muscle | MFH with > 95% necrosis |
| 454.001 | 75% muscle, fat | ** |
| 454.002 | 75% muscle, fat and fibrous tissue | Leiomyosarcoma |
| 462.001 | 90% muscle, fibrous tissue | Sarcoma NOS[a] with > 80% necrosis |
| 462.002 | 90% muscle, fibrovascular tissue | Sarcoma NOS[a] with > 80% necrosis |
| 464.001 | Muscle | PNST |
| 464.002 | Muscle | PNST |
| 475.001 | Muscle | MFH |
| 475.002 | Muscle | * |
| 482.001 | Muscle | * |
| 482.002 | Muscle | * |
| 485.001 | — | Myxoid liposarcoma |
| 485.002 | Muscle | Myxoid liposarcoma |
| 485.003 | Muscle | — |
| 486.001 | 75% muscle, fibrous tissue | Myxoid liposarcoma |
| 486.002 | Muscle | Myxoid liposarcoma |
| 488.001 | Muscle | Liposarcoma |
| 488.002 | Muscle | Liposarcoma |
| 554.001 | Muscle | MFH |
| 554.002 | Muscle | MFH |

[a] NOS, not otherwise specified; —, not included in the analysis due to poor spectral quality; *Spectra were put in the test set. The tumor bank report and SCS classifier indicated the presence of tumor but histopathological assessment of the tissue subjected to MRS did not. **Spectrum was put in the test set. Tumor bank report indicated tumor but both the SCS and histopathology did not.

90° pulse at 8.0 μs; number of scans, 256 or 640 depending on the size of the sample; spectral width = 5000 Hz; recycle delay = 2.41s; and time domain data points, 4K. Immediately following the MRS experiments, all samples were fixed in 10% buffered formalin and submitted for histopathological evaluation. The MR spectra were archived in a Silicon Graphics Computer, and analyzed by the statistical classification strategy as detailed below.

The resonance areas (intensities) were also determined for the following seven spectral regions, using standard Bruker integration routines. The integration limits (ppm) for these regions were 3.94–3.88, 3.44–3.38, 3.30–3.13, 3.07–2.90, 2.54–1.83, 1.83–1.05 and 1.05–0.61. The ratios of these values for both normal and malignant specimens were determined and compared using the Student *t*-test ($P < 0.05$; Microsoft Excel 97). For multiple specimens from the same patient, average values were used in the comparison. Peak area ratios are reported as mean ± standard deviation.

For the statistical classification strategy, the MR spectra were partitioned randomly into a *training set* (15 normal, 15 malignant) and a *test set* (12 normal, 12 malignant) before being subjected to the analysis. The classifier was developed using the training set and its accuracy validated on the test set. Each magnitude spectrum was aligned on the reference peak (*p*-aminobenzoic acid) at 6.81 ppm and normalized by dividing every data point by the total spectral area. The 0.5–4.0-ppm region of each spectrum was selected in order to minimize the spectral artifact created by suppression of the water peak at 4.7 ppm. This region of 550 data points was divided into 110 equal subregions by averaging five consecutive data points. An optimal region selection genetic algorithm[28] was employed to determine the regions of interest. The classification was performed using linear discriminant analysis (LDA) with the leave-one-out (LOO) method on the training set.

### Results

Figure 1 shows representative spectra from malignant fibrous histiocytoma (MFH) and an adjacent normal mesenchymal tissue. The major resonances of potential diagnostic interest are labeled as indicated. Spectral resonances were assigned via two-dimensional COSY spectra and by comparison with chemical shift values of standard substances and literature values.[18,19,29] The two resonances indicated for creatine, at 3.04 and 3.93 ppm, are due to the $-CH_3$, and

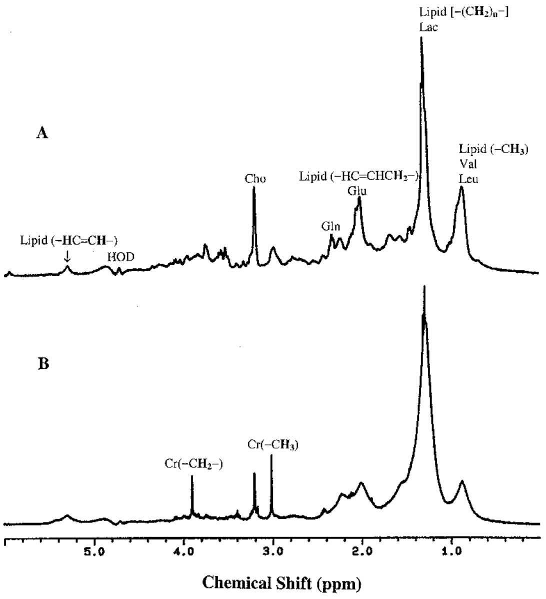

**Fig. 1.** *Proton MR spectra (360 MHz, 25°C) of soft tissue specimens: (A) malignant fibrous histocytoma, (B) adjacent normal tissue from the same subject. Abbreviations: Chos, choline-containing compounds; Cr, creatines; Gln, glutamine; Glu, glutamic acid; HOD, deuteriated water; Leu, leucine; Val, valine. Assignments do not imply that these are the only substances contributing to a particular peak.*

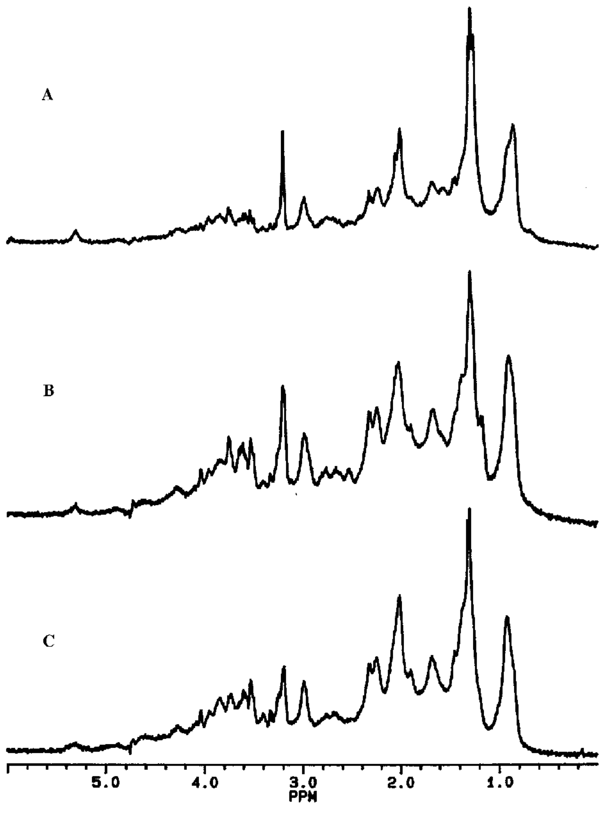

**Fig. 2.** *Proton MR spectra (360 MHz, 25°C) of soft tissue sarcoma of different histological types: (A) malignant fibrous histocytoma, (B) leiomyosarcoma, and (C) fibrosarcoma. Spectral peak assignments are the same as in Fig. 1.*

$-CH_2-$ groups of the compound, respectively. There are notable differences between the two spectra in many of the resonances, including those from choline and creatine. For example, higher levels of creatine and lower levels of choline-containing metabolites are seen in the spectra of the normal tissue compared to that of the tumor. However, there may also be other potentially useful differences that are too difficult to discriminate by visual inspection, and thus there is the need for the computerized statistical classification approach. It is worthwhile to note here that spectra obtained from multiple pieces of a specimen (normal or tumor) were very similar.

Figure 2 shows three representative spectra from soft tissue sarcomas of different histological types: MFH, leiomyosarcoma and fibrosarcoma. The rationale for showing this figure is to underscore the fact that despite numerous small differences, the spectra have a similar overall appearance, reflective of their common malignant property.

Besides performing the SCS-based analysis, an attempt was also made to determine whether some of the spectral intensity ratios could serve as useful diagnostic markers, since this method had been used in earlier studies.[30,31] The mean values of the ratios (for both normal and malignant) that showed statistically significant differences are indicated in Table 2, as well as the *P* values of the statistical comparison. Although not listed in Table 2, the sensitivity and specificity for detecting soft tissue malignancy were also determined for all these spectral ratios. As can be seen in Table 2, the best *P* value was obtained for the 0.9/2.0 spectral intensity ratio resulting in a sensitivity of 100% and a specificity of 92.8%. It is worthwhile to note here that there was no test set in this conventional analysis.

An optimal set of six subregions was selected by our algorithm as having discriminating potential. These subregions include resonances of choline, creatine, glutamine, glutamic acid, and lipids. The other subregion selected by the algorithm was at ~2.63 ppm, for which we currently have no biochemical attribution.

The SCS-based classifier resulted in good classification accuracy for both the training and the test sets. The sensitivity and specificity of the technique for detecting cancer are indicated in Table 3. Note here that there was a disagreement between the initial diagnoses provided by the tumor bank and the ones obtained from histopathological examination of the tissue specimens subjected to MRS. There was a better agreement of the SCS results with the former. However, we have used the histopathological data to calculate our classification accuracy.

These preliminary results show that $^1H$ MRS, combined with the appropriate statistical classification strategy, gives high overall accuracy, and could potentially be used to distinguish between normal mesenchymal tissue and malignant soft tissue sarcomas.

**Table 2.** *Comparison of mean values of $^1H$ MR spectral area ratios in soft tissue sarcoma*

| Spectral Area Ratio | Normal* Mean ± S.D. | Malignant* Mean ± S.D. | *P* value |
|---|---|---|---|
| 0.9/2.0 | 0.49 ± 0.07 | 0.73 ± 0.11 | 0.0000003 |
| 1.3/2.0 | 4.47 ± 0.26 | 3.31 ± 1.17 | 0.002 |
| 0.9/1.3 | 0.11 ± 0.02 | 0.25 ± 0.11 | 0.0001 |
| 3.4/3.9 | 0.42 ± 0.16 | 0.87 ± 0.59 | 0.01 |
| 3.2/3.9 | 3.49 ± 0.97 | 14.20 ± 9.10 | 0.005 |

*Although the total number of spectra used was 54 (27 normal, 27 malignant), the numbers of the averaged values included in the analysis were 13 and 14 for the normal and malignant specimens respectively.

**Table 3.** *Results[a] of the SCS-based classification of $^1H$ MR spectra in the diagnosis of soft tissue sarcoma*

| | Classification accuracy | Sensitivity | Specificity |
|---|---|---|---|
| Training set | 100 | 100 | 100 |
| Test set | 83 | 92 | 76 |
| Overall | 93 | 100 | 88 |

[a]Based on the histopathological assessment of the tissue subjected to MRS.

### Discussion

The $^1H$ MR spectra from soft tissue sarcoma are qualitatively similar to those of other tissue types, with many of the common resonances present. However, there exist some spectral differences between soft tissue sarcomas and other tissue specimens investigated in our laboratory. We do not normally observe a singlet at 3.93 ppm attributable to the $-CH_2-$ of creatine in normal and tumor tissue specimens from colon, prostate, cervix, head and neck. The reason for this is not evident at the present time. However, we do observe the other creatine resonance (due to $-CH_3$) in all the above normal tissue specimens, including those coming from soft tissue.

Comparing the results obtained by the conventional analysis of spectral ratios and the SCS-based method illustrates three significant points. First, unlike the SCS, the conventional peak area ratio analysis does not generally use a test set. All the data are treated as the training set. Validating the accuracy of the classifier on a test set is essential for developing a robust and reliable diagnostic classifier for clinical use. Second, overall classification accuracy is higher for the SCS-based classifier than for the conventional analysis. Third, the conventional analysis looks at some preselected resonances/ratios based on some prior knowledge, whereas such prior knowledge is not required for applying the SCS.

The diagnostic spectral regions selected by our algorithm are consistent with findings in other types of tumors. It is worth emphasizing that these regions were selected by the algorithm without any prior input from the user. The finding of higher choline metabolite levels indicates an increase in cell proliferation and membrane biosynthesis in tumors. Similar results have been obtained for prostate, brain, breast, and colon tumors.[22,23,30–32] MRS studies done to date on soft tissue sarcoma show higher levels of triglycerides in normals compared to tumors. Alterations in cellular lipid composition have been found to play an important role in determining the metastatic behaviour of tumor cells.[33] The degree of fatty acyl unsaturation is believed to be an important determinant of the metastatic potential of soft tissue sarcomas.[17] The significantly higher 0.9/1.3 ratio ($P = 0.0001$) found for tumors in our study is consistent with this. The reduction of creatine, indicative of increased energy metabolism, is also observed in both brain tumors and malignant prostate tissue. The selection of glutamic acid as one of the discriminatory regions is also consistent with other findings. Glutamic acid levels have been found to be significantly higher in cancers of colon and stomach.[34]

One of the common features of soft tissue sarcomas such as liposarcomas has been an intense broad lipid resonance. That is why Millis *et al.* proposed the use of magic angle spinning (MAS) to resolve the broad spectral resonances and obtain useful information.[18,19] Although such a technique, without any doubt, improves the spectral resolution, it adds complexity of measurement and sample preparation and lengthens measurement time. Moreover, a potential disadvantage of the MAS technique is that it adds many resonances to an already crowded spectrum, possibly decreasing the discriminating power. The present approach is simple to use, robust, and nonsubjective, since it makes use of a computerized statistical classification strategy. One of the advantages of MAS, however, has been in the resolution of the resonance at 3.21 ppm due to choline-containing compounds. This broad peak consists of resonances from choline, phosphocholine, glycerophosphocholine, and phosphatidylcholine. Some of these resonances play a larger role than others in the development and growth of the tumor and, thus, the resolution of these resonances and their relative contribution may be necessary for a thorough understanding of the biochemical changes associated with malignancy.

The fact that we were able to include tumors with different histological types and still obtain high accuracy indicates that the spectral differences between normal and malignant specimens are much larger than those among the different histological types/subtypes. Correlations of spectral features with the degree of differentiation and histological types/subtypes would require a much larger sample size.

The next step in our study is to test whether MRS, combined with a SCS-based classifier, can differentiate between benign and malignant tumors, a clinically more relevant issue. Once the spectral patterns of normal, benign, malignant (different types) are identified *ex vivo* using the SCS, such information will be used in an *in vivo* setting. Ultimately, the objective is the development of a *non-invasive and non-subjective technique* to accurately diagnose the histological type, the grade and the stage of soft tissue sarcoma. Besides improving our ability to prognosticate, it should also be helpful in identifying patients who could benefit from adjuvant therapy. The fact that 50% of soft tissue sarcomas tend to arise in the extremities, i.e., MR-accessible sites, and remain localized to the region of origin in the majority of patients, makes them ideal targets for localized *in vivo* MR spectroscopy. Potentially, MRS *in vivo* can be a useful tool in the diagnosis, prediction of prognosis, choice of therapy, outlining the exact margin of tumors, and monitoring of treatment. The types of sequences and parameters used for different purposes in MRI of soft tissue sarcoma are discussed at length by Hanna and Fletcher.[9] For spectroscopy, one would need to use phased array coils and the PRESS sequence for spectroscopic localization[35] to obtain good quality spectra from the volume of interest. Although other coils can be used for the same purpose, the use of phased array coils is recommended to maximize the signal. *In vivo* $^{31}$P MR spectroscopy has already been attempted in soft tissue sarcomas and the results have been promising.[12] In fact, soft tissue sarcoma is one of the few types of cancer that is currently being investigated in an ongoing multi-institutional trial involving nine centers of localized *in vivo* $^{31}$P MR spectroscopy in human cancer research.[36] A similar effort should also be directed towards the use of $^{1}$H MRS. Since both MRI and MRS can be performed using the same instrument, this could easily be combined with a regular MRI examination.

### Acknowledgements

We would like to thank Dr. Irene Andrulis of Mount Sinai Hospital (Toronto) for her help in obtaining the tissue specimens. We are very grateful to the Canadian National Sarcoma Group for letting us use tissue specimens from their bank. This work was presented in part at the following meetings: International Society of Magnetic Resonance in Medicine (Glasgow, Scotland, May 2001) and American Society of Clinical Oncology (San Francisco, California, May 2001).

### References

1. Singer S, Corson JM, Gonin R, *et al.* Prognostic factors predictive of survival and local recurrence for extremity soft tissue sarcoma. *Ann Surg* 1994; 219: 165–73.
2. Singer S. New diagnostic modalities in soft tissue sarcoma. *Semin Surg Oncol* 1999; 17: 11–22.
3. Alvegard TA, Berg NO. Histopathology peer review of high-grade soft tissue sarcoma: The Scandinavian sarcoma group experience. *J Clin Oncol* 1989; 7: 1845–52.
4. Coindre JM, Trojani M, Contesso G, *et al.* Reproducibility of a histopathologic grading system for adult soft tissue sarcoma. *Cancer* 1986; 58: 306–9.
5. Lewis JJ, Brennan MF. Soft tissue sarcomas. *Curr Probl Surg* 1996; 33: 820–72.
6. Levine EA. Prognostic factors in soft tissue sarcoma. *Semin Surg Oncol* 1999; 17: 23–32.
7. Heslin MJ, Smith JK. Imaging of soft tissue sarcoma. *Surg Oncol Clin North Am* 1999; 8: 91–107.
8. Massengill AD, Seeger LL, Eckardt JJ, *et al.* The role of plain radiography, computed tomography, and magnetic resonance imaging in sarcoma evaluation. *Hematol Oncol Clin North Am* 1995; 9: 571–604.
9. Hanna SL, Fletcher BD. MR Imaging of malignant soft tissue tumors. *Magn Reson Imaging Clin North Am* 1995; 3: 629–50.
10. Varma DGK, Jackson EF, Pollock RE, *et al.* Soft-tissue sarcoma of the extremities: MR appearance of post-treatment changes and local recurrences. *Magn Reson Imaging Clin North Am* 1995; 3: 695–712.
11. Negendank WG, Crowley MG, Ryan JR, *et al.* Bone and soft-tissue lesions: diagnosis with combined H-1 MR imaging and P-31 MR spectroscopy. *Radiology* 1989; 173: 181–6.
12. Negendank WG. MR spectroscopy of musculoskeletal soft-tissue tumors. *Magn Reson Imaging Clin North Am* 1995; 3: 713–25.
13. Shinkwin MA, Lenkinski RE, Daly JM, *et al.* Integrated magnetic resonance imaging and phosphorus spectroscopy of soft tissue tumors. *Cancer* 1991; 67: 1849–58.
14. Sostman HD, Prescott DM, Dewhirst MW, *et al.* MR imaging and spectroscopy for prognostic evaluation in soft-tissue sarcomas. *Radiology* 1994; 190: 269–75.
15. Redmond OM, Bell E, Stack JP, *et al.* Tissue characterization and assessment of musculoskeletal tumors by *in vivo* 31P magnetic resonance spectroscopy. *Magn Reson Med* 1992; 27: 226–37.
16. Singer S, Millis K, Souza K *et al.* Correlation of lipid content and composition with liposarcoma histology and grade. *Ann Surg Oncol* 1997; 4: 557–63.
17. Singer S, Sivaraja M, Souza K, *et al.* $^{1}$H-NMR detectable fatty acyl chain unsaturation in excised leiomyosarcoma correlate with grade and mitotic activity. *J Clin Invest* 1996; 98: 244–50.
18. Millis KK, Mass WE, Cory DG, *et al.* Gradient, high-resolution, magic-angle spinning nuclear magnetic resonance spectroscopy of human adipocyte tissue. *Magn Reson Med* 1997; 38: 399–403.
19. Millis K, Weybright P, Campbell N, *et al.* Classification of human liposarcoma and lypoma using *ex vivo* proton nmr spectroscopy. *Magn Reson Med* 1999; 41: 257–67.
20. Mountford CE, Somorjai RL, Malycha P, *et al.* Diagnnosis and prognosis of breast cancer by magnetic spectroscopy of fine-needle aspirates analysed using a ststistical classification strategy. *Br J Surg* 2001; 88: 1234–40.
21. Hahn P, Smith ICP, Leboldus L, *et al.* The classification of benign and malignant human prostate tissue by multivariate analysis of $^{1}$H magnetic resonance spectra. *Cancer Res* 1997; 57: 3398–401.
22. Menard C, Smith ICP, Somorjai RL, *et al.* Magnetic resonance spectroscopy of the malignant prostate gland after radiotherapy: a histopathological study of diagnostic validity. *Int J Radiat Oncol Biol Phys* 2001; 50: 317–23.

23. Bezabeh T, Smith ICP, Krupnik E, *et al.* Diagnostic potential for cancer via $^{1}$H magnetic resonance spectroscopy of colon tissue. *Anticancer Res* 1996; 16: 1553–8.
24. Somorjai RL, Dolenko B, Nikulin AK, *et al.* Classification of $^{1}$H MR spectra of human brain neoplasms: the influence of preprocessing and computerized consensus diagnosis on classification accuracy. *J Magn Reson Imaging* 1996; 6: 437–44.
25. Wallace JC, Raaphorst GP, Somorjai RL, *et al.* Classification of $^{1}$H MR spectra of biopsies from untreated and recurrent ovarian cancer using linear discriminant analysis. *Magn Reson Med* 1997; 38: 569–76.
26. Somorjai RL, Nikulin AE, Pizzi N, *et al.* Computerized consensus diagnosis: a classification strategy for the robust analysis of MR spectra. I. Application to $^{1}$H spectra of thyroid neoplasms. *Magn Reson Med* 1995; 33: 257–63.
27. Kuesel AC, Kroft T, Saunders JK, *et al.* A simple procedure for obtaining high-quality NMR spectra of semi quantitative value from small tissue specimens: cervical biopsies. *Magn Reson Med* 1992; 27: 340–55.
28. Nikulin AE, Dolenko B, Bezabeh T, *et al.* Near-optimal region selection for feature space reduction: novel-preprocessing methods for classifying MR spectra. *NMR Biomed* 1998; 11: 209–17.
29. Sze DY, Jardetzky O. Characterization of lipid composition in stimulated human lymphocytes by $^{1}$H-NMR. *Biochim Biophys Acta* 1990; 1054: 198–206.
30. Lean CL, Newland RC, Ende DA, *et al.* Assessment of human colorectal biopsies by $^{1}$H MRS: correlation with histopathology. *Magn Reson Med* 1993; 30: 525–33.
31. Mackinnon WB, Barry PA, Malycha PL, *et al.* Fine-needle biopsy specimens of benign breast lesions distinguished from invasive cancer ex vivo with proton MR spectroscopy. *Radiology* 1997; 204: 661–6.
32. Daly PF, Lyon RC, Faustino PJ, *et al.* Phospholipid metabolism in cancer cells monitored by $^{31}$P NMR spectroscopy. *J Biol Chem* 1987; 31: 14875–78.
33. Dahiya R, Boyle B, Goldberg BC, *et al.* Metastasis-associated alterations in phospholipids and fatty acids of human prostatic adenocarcinoma cell lines. *Biochem Cell Biol* 1992; 70: 548–54.
34. Okada A, Takehara H, Yoshida K, *et al.* Increased aspartate and glutamate levels in both gastric and colon cancer tissues. *Tokushima J Exp Med* 1993; 40: 19–25.
35. Bottomley PA. Spatial localization in NMR spectroscopy. *Ann NY Acad Sci* 1987; 508: 333-48.
36. Arias-Mendoza F, Brown TR, Charles HC, *et al.* Methodological standardization for a multi-institutional *in vivo* trial of localized $^{31}$P MR spectroscopy in human cancer research. *Proc Int Soc Mag Reson Med* 1999; 7: 1585 (Abstr).
